# Development and Pilot Validation of an Age-Friendly City Assessment Tool Based on Older Adults’ Perspectives in a Semi-Urban Community

**DOI:** 10.3390/ijerph23030287

**Published:** 2026-02-26

**Authors:** Autchariya Punyakaew, Pich Karakate, Tanaporn Nukeaw, Thanaporn Saopasee, Supawadee Putthinoi

**Affiliations:** Department of Occupational Therapy, Faculty of Associated Medical Sciences, Chiang Mai University, 110 Intawaroros Rd., Sripoom, Chiang Mai 50200, Thailand; autchariya.punyakaew@cmu.ac.th (A.P.); pich_karakate@cmu.ac.th (P.K.); tanaporn_n@cmu.ac.th (T.N.); thanaporn_sao@cmu.ac.th (T.S.)

**Keywords:** age-friendly cities, older adults, semi-urban communities, psychometric assessment, community environment, aging

## Abstract

**Highlights:**

**Public health relevance—How does this work relate to a public health issue?**

- Population aging and urbanization require age-friendly community environments to support healthy aging and functional independence.
- Existing age-friendly city assessments often rely on administrative or expert evaluations, which may overlook older adults’ lived experiences in semi-urban settings.

**Public health significance—Why is this work of significance to public health?**

- This study develops and pilot-validates a perception-based Age-Friendly City assessment tool tailored to semi-urban communities in a low- and middle-income country context.
- The instrument demonstrates strong content validity and excellent preliminary reliability, supporting its use in community-based public health assessment and planning.

**Public health implications—What are the key implications or messages for practitioners, policy makers and/or researchers in public health?**

- The tool provides local authorities and public health practitioners with a practical method to identify environmental facilitators and barriers affecting older adults’ participation and well-being.
- Findings support the importance of incorporating older adults’ perspectives into age-friendly policies and interventions to promote inclusive, healthy, and sustainable communities.

**Abstract:**

Background: Age-friendly city (AFC) initiatives are widely promoted to support healthy aging; however, most existing AFC assessments rely on administrative or expert-driven evaluations that primarily reflect institutional perspectives. These approaches may overlook how age-friendly characteristics are experienced by older adults—the population most directly affected by community environments—particularly in semi-urban settings. This study aimed to develop and conduct a preliminary psychometric evaluation of an AFC assessment tool based on older adults’ perspectives. Methods: A Research and Development (R&D) design was employed. The instrument was conceptually grounded in the World Health Organization Age-Friendly Cities framework and adapted from a governmental checklist through item reformulation and contextual modification for semi-urban application in Thai setting. Content validity was examined by an expert panel using the Index of Item–Objective Congruence (IOC). Preliminary internal consistency reliability testing was conducted with a small purposive sample of older adults. The refined instrument was then pilot-tested with an independent sample of community-dwelling older adults to evaluate feasibility and descriptive response patterns. Internal consistency reliability was assessed using Cronbach’s alpha, and descriptive analyses were performed across domains and subdomains. Results: The finalized instrument comprised 52 items across three domains and eight subdomains. Content validity was strong, with IOC values ranging from 0.80 to 1.00. Preliminary reliability testing demonstrated high internal consistency (Cronbach’s alpha = 0.97), indicating suitability for pilot use while suggesting potential item redundancy. Pilot responses showed predominantly high perceived age-friendliness, with moderate scores in selected subdomains. Conclusions: The AFC Assessment Tool demonstrated strong preliminary psychometric properties and practical feasibility for use among community-dwelling older adults in semi-urban settings. By incorporating older adults’ perspectives, the tool provides a context-sensitive approach that complements existing administrative and objective assessments. Further validation using larger and more diverse samples is needed to establish construct validity, confirm dimensional structure, and strengthen applicability in public health and environmental gerontology research.

## 1. Introduction

Population aging is a defining global shift that places increasing pressure on health systems and urban planning to support autonomy and quality of life in later adulthood [1]. In response, the World Health Organization (WHO) introduced the Active Ageing policy framework [2] and the Global Age-Friendly Cities (AFC) initiative [3], emphasizing inclusive environments that enhance older adults’ well-being across eight domains: outdoor spaces and buildings, transportation, housing, social participation, respect and social inclusion, civic participation and employment, communication and information, and community support and health services. Although the term “age-friendly city” originates from the WHO framework, its underlying principles—environmental accessibility, social inclusion, civic participation, and supportive services—apply across diverse community types, not only large metropolitan areas. Increasingly, the WHO AFC framework has been adapted for rural and semi-urban settings, where environmental configurations and service structures differ but the overarching goal of promoting active aging remains consistent. In the present study, the term is used conceptually to reflect alignment with the WHO domains while acknowledging the semi-urban characteristics of the study area situated along the rural–urban continuum.

The AFC framework has become a cornerstone for guiding global and national aging policies. In Thailand, it is incorporated into the National Plan for Older Persons (2023–2040) and related policies of the Ministry of Social Development and Human Security and the Ministry of Public Health [4]. These initiatives aim to improve equitable access to healthcare, appropriate housing, transportation, and opportunities for community participation among older adults. However, the translation of national-level strategies into local action remains inconsistent. Disparities persist between urban and semi-urban contexts in terms of environmental accessibility, social participation, and community readiness to support aging in place. Although AFC assessments are often conducted using checklists completed by municipal authorities, such top–down evaluations may not adequately capture older adults’ perceptions or lived experiences—particularly in semi-urban communities that combine rural and urban features.

Most existing AFC assessment instruments were developed in high-income, urban contexts. When applied directly to semi-urban settings in low- and middle-income countries, these tools may lack contextual sensitivity to local living patterns, resource constraints, and sociocultural norms [5]. Accordingly, there is a critical need for tools that reflect older adults’ lived experiences within specific community contexts.

The study developed the Elderly Friendly Urban Spaces Questionnaire using both qualitative content analysis-grounded theory for item development and quantitative psychometric validation involving structural validity, split-half analysis, and Cronbach’s Alpha to confirm reliability [6]. Consequently, there is a growing recognition that the assessment of age-friendliness must move beyond direct adoption of international models toward contextual adaptation and cultural validation.

Thailand is undergoing a rapid demographic transition, highlighting the need for empirical studies that examine age-friendly environments across diverse community contexts. In this study, Nong Phueng Subdistrict, Chiang Mai Province, is classified as a semi-urban area and operationally defined as a local administrative unit (Tambon) situated along the rural–urban continuum. Semi-urban areas are typically characterized by transitional features, including moderate population density 1500–3000 persons/km^2^, evolving local infrastructure, and mixed economic activities encompassing both agricultural and service sectors [7]. These characteristics reflect population patterns influenced by both rural and urban contexts and are consistent with interdisciplinary definitions of semi-urban environments as hybrid zones between predominantly urban and rural areas [8].

Applying the AFC framework in such settings requires contextual adaptation rather than direct adoption of the WHO conceptual model [9,10]. Cross-national research demonstrates substantial variation in age-friendly indicators across urban and rural environments, especially in low- and middle-income countries, reflecting differences in infrastructure, service availability, and social organization [11]. Semi-urban areas thus represent transitional spaces where such variations are particularly relevant. Nong Phueng Subdistrict provides a meaningful context for operationalizing the AFC framework and for developing an assessment tool that captures older adults’ perceptions of their community environment.

From an occupational therapy perspective, the Person–Environment–Occupation–Performance (PEOP) model underscores the dynamic interaction between individuals and their environments in shaping daily participation [12]. This framework highlights the importance of examining environmental and social factors that may facilitate or constrain engagement. A perception-based AFC assessment tool aligns conceptually with the PEOP model by adopting a client-centered approach that incorporates older adults’ perspectives as users of their community environments.

Although several age-friendly assessment instruments exist internationally, many were designed either for urban areas in high-income countries or for completion by municipal authorities. Few tools have been adapted for semi-urban communities in low- and middle-income countries or designed to directly capture older adults’ lived experiences. Existing instruments also tend to emphasize structural or policy-level indicators rather than subjective perceptions.

The present study contributes to the literature by combining contextual and conceptual innovation. First, the tool is adapted specifically for semi-urban settings characterized by hybrid rural–urban features. Second, it is designed for direct administration to older adults using a perception-based Likert scale rather than administrative checklists. Third, the tool is developed in a low- and middle-income country context where infrastructure and service systems differ substantially from high-income urban environments. Finally, the instrument is conceptually grounded in the PEOP model, providing a theoretical basis for interpreting environmental influences on participation.

Accordingly, the objective of this study was to develop and conduct a preliminary psychometric evaluation of the AFC Assessment Tool, including assessment of content validity and internal consistency in a pilot sample. The tool was designed to assess age-friendly characteristics in semi-urban Thai communities from the perspective of older adults and to provide a structured, contextually relevant instrument for community-level assessment and planning.

## 2. Methods

### 2.1. Study Location

Nong Phueng Subdistrict is located in Saraphi District, Chiang Mai Province, Northern Thailand. The subdistrict is situated approximately 10 km southeast of Chiang Mai city and represents a semi-urban community undergoing gradual urbanization. The area comprises eight villages—Ban Nong Phueng, Ban Chiang Saen, Ban Don Chin, Ban Nong Phueng Tai, Ban Pa Khae Yong, Ban Kong Sai, Ban Pa Ket Thii, and Ban San Khue—with a total population characterized by a high proportion of older adults.

Administratively, Nong Phueng Subdistrict is governed by a Subdistrict Administrative Organization and supported by a subdistrict health-promoting hospital, village health volunteers, and active elderly clubs. The community exhibits a mix of traditional rural lifestyles and emerging urban infrastructure, including transportation networks, public spaces, and community services. This transitional context makes Nong Phueng Subdistrict an appropriate setting for developing and pilot-testing an Age-Friendly City assessment tool, as it reflects environmental and social conditions commonly encountered in semi-urban communities in Thailand.

### 2.2. Study Design

This study employed a Research and Development (R&D) design focused on instrument development and pilot validation, without employing a mixed-methods research framework. Data collection took place between October 2024 and February 2025.

The R&D process comprised three sequential phases:

(1) Instrument development and content validation;
(2) Preliminary internal consistency reliability testing;
(3) Pilot testing to examine feasibility and descriptive response patterns.

Analyses were limited to expert-based content validity and internal consistency due to the pilot sample size. Structural and construct validity analyses were not conducted at this stage.

### 2.3. Phase 1: Instrument Development and Content Validation

#### 2.3.1. Item Generation and Instrument Adaptation

An initial pool of items was generated based on the World Health Organization (WHO) Age-Friendly Cities framework. A Thai-language AFC operational checklist developed by the Department of Health, Ministry of Public Health, Thailand, originally intended for use by local administrative authorities, served as the primary reference source [13]. The tool was conceptually structured into three domains and eight subdomains, aligning theoretically with the WHO Age-Friendly Cities framework. It is important to note that this structure is derived from existing theory rather than empirical factor analysis.

Although the overall domain structure was retained in alignment with the WHO age-friendly framework to preserve conceptual comparability, contextual adaptation was implemented at the item level. Items were operationalized to reflect environmental characteristics specific to semi-urban communities situated along the rural–urban continuum. These adaptations included references to mixed formal–informal transportation systems, evolving housing configurations, localized service accessibility, and community-based social networks that differ from those typically observed in metropolitan settings.

To ensure suitability for direct administration to older adults, checklist items were reformulated as self-report statements. In addition, the response format was adapted from a binary (Yes/No) checklist to a five-point Likert scale to capture graded perceptions of age-friendly community characteristics, consistent with recommended practices for patient- and population-reported outcome measures [14,15]. Items were rated on a six-point Likert scale ranging from 0 (very low/not supportive) to 5 (very high/highly supportive). An even-numbered response format was intentionally selected to reduce central tendency bias and avoid a neutral midpoint, thereby encouraging respondents to express a directional perception regarding environmental supportiveness. This format has been recommended in perception-based assessments to enhance discriminatory power and reduce ambivalent responding.

#### 2.3.2. Expert Review and Content Validity

The initial version of the questionnaire consisted of 56 items. Content validity was evaluated by a panel of five experts, including three geriatric occupational therapists, one public health academic, and one family medicine physician with experience in geriatric care. Experts independently assessed each item for relevance, clarity, and representativeness in relation to the WHO AFC domains.

Item-level content validity was quantified using the Index of Item–Objective Congruence (IOC). Items with IOC values below 0.50 were revised or removed based on expert recommendations, consistent with established guidelines for content validation in instrument development.

#### 2.3.3. Cognitive Debriefing with Older Adults

Cognitive debriefing was conducted between October and November 2024 with five community-dwelling older adults to assess linguistic clarity and cultural appropriateness. This procedure was implemented as a methodological refinement step within the R&D process rather than as a standalone qualitative research design.

Using a structured think-aloud approach, participants reviewed each item sequentially and provided feedback on clarity, interpretation, and confidence in selecting response options. Items that generated uncertainty, ambiguous interpretation, or hesitation among at least three participants were flagged for revision. Revised wording was discussed and re-evaluated within the group to achieve consensus. Modifications primarily involved simplifying wording, clarifying contextual references, and adjusting phrasing to better reflect everyday community experiences. Modifications primarily involved simplifying wording, clarifying contextual references, and adjusting phrasing to better reflect everyday community experiences. This iterative refinement process aligns with established guidelines for cognitive interviewing and cross-cultural adaptation of self-report instruments [14,15]. Minor revisions were completed prior to progression to Phase 2.

### 2.4. Phase 2: Preliminary Internal Consistency Reliability Testing

#### 2.4.1. Participants and Procedure

Preliminary internal consistency was assessed using a purposive sample of 10 community-dwelling older adults aged 60 years and above recruited from Nongpakrang Municipality, Mueang District, Chiang Mai Province, Thailand. This phase aimed to examine initial item performance and feasibility and was exploratory in nature; it was not intended to provide definitive estimates of reliability.

Participants demonstrated no evidence of cognitive impairment, as confirmed by scores above the education-adjusted cut-off points on the Mental State Examination T10 (MSET10) [16]. All participants were able to comprehend the questionnaire items and provided written informed consent prior to enrollment. Participants in Phase 2 were not included in subsequent pilot testing.

#### 2.4.2. Data Analysis

Internal consistency reliability of the refined instrument was evaluated using Cronbach’s alpha coefficients. This phase aimed to identify potential internal consistency issues prior to pilot feasibility testing and was not intended to establish definitive psychometric properties, in line with methodological recommendations for early-stage instrument development [17,18,19].

### 2.5. Phase 3: Pilot Testing for Feasibility

#### 2.5.1. Participants

Pilot testing was conducted using an independent sample of community-dwelling older adults aged 60 years and above residing in Nong Phueng Subdistrict, Saraphi District, Chiang Mai Province, Thailand. Participants included in this phase were not involved in the preliminary reliability testing conducted in Phase 2.

Eligibility criteria included residence in the subdistrict for at least six months, absence of cognitive impairment verified by passing the MSET10 with education-adjusted cut-off scores [16], ability to communicate in Thai and comprehend the questionnaire items, and provision of written informed consent.

#### 2.5.2. Sampling Procedure and Sample Size

A purposive sampling technique was employed. An initial target of six participants from each of the eight villages was planned, yielding a target sample of 48 participants. Three individuals withdrew or were excluded due to incomplete participation, resulting in a final sample of 45 participants. The sample size was considered appropriate for pilot testing aimed at examining feasibility, response patterns, and preliminary internal consistency rather than conducting factor-analytic validation, consistent with methodological guidance for early-stage instrument development and psychometric research [17,18,19,20].

#### 2.5.3. Data Analysis

Descriptive statistical analyses were conducted to summarize participant characteristics and AFC domain scores. Frequencies, percentages, means, and standard deviations were used to describe perceived age-friendly characteristics across domains. No inferential analyses or factor analyses were performed, consistent with the pilot nature of the study.

## 3. Results

The results are presented to demonstrate the feasibility, preliminary performance, and descriptive response patterns of the developed AFC Assessment Tool when applied in a semi-urban community context. As this study represents the pilot phase of an instrument development process, the findings are intended to illustrate the application of the tool and its capacity to capture variation in perceived age-friendly characteristics, rather than to provide definitive community-level evaluation or comparative benchmarking.

### 3.1. Instrument Structure

The AFC assessment tool was structured into three domains and eight subdomains based on theoretical alignment with the WHO age-friendly framework, as presented in Table 1. The Physical and Environmental domain comprises three subdomains: Outdoor Spaces and Buildings, Transportation Systems, and Housing. The Social domain includes Social Participation, Respect and Social Inclusion, and Civic Participation and Employment. The Support and Services domain encompass Communication and Information and Community Support and Health Services.

Each item was designed to capture older adults’ perceptions of age-friendly community characteristics and was rated on a 6-point Likert scale ranging from 0 to 5, with higher scores indicating more favorable perceptions. This scoring structure allows for graded assessment of perceived accessibility, adequacy, and supportiveness of environmental and community features relevant to older adults.

### 3.2. Item Refinement and Content Validity

Item refinement was conducted as part of the instrument development process based on expert review and content validity analysis. As summarized in Table 2, the initial version of the AFC assessment tool consisted of 56 items across three domains and eight subdomains. Following content validation using the IOC, four items were removed due to IOC values below 0.50 or conceptual overlap with other items within the same subdomain.

In the Physical and Environmental domain, one item each was removed from Outdoor Spaces and Buildings, Transportation Systems, and Housing due to inadequate IOC values and redundancy. In the Social domain, one item was removed from Civic Participation and Employment, while items in Social Participation and Respect and Social Inclusion were retained with minor wording revisions to improve clarity and readability. Within the Support and Services domain, all items in Communication and Information and Community Support and Health Services were retained, with refinements made to enhance linguistic clarity and alignment with operational definitions.

As a result of this refinement process, the finalized instrument comprised 52 items, with item-level IOC values ranging from 0.80 to 1.00, indicating strong content validity across all domains and subdomains.

All domains and subdomains were derived from the WHO’s AFC Framework [3]. Item contents were adapted from the Thai AFC checklist developed by the Department of Health, Ministry of Public Health, Thailand (2023) [13]. Item refinement was guided by expert content validation using the IOC and cognitive debriefing with older adults.

### 3.3. Internal Consistency Reliability

The overall internal consistency of the instrument was high (Cronbach’s α = 0.97). Subscale analyses demonstrated similarly strong reliability across the three domains. The Physical & Environmental domain yielded a Cronbach’s alpha of 0.95, the Social domain demonstrated an alpha of 0.93, and the Support & Services domain showed an alpha of 0.97. These findings indicate high internal consistency at the domain level. However, the overall alpha coefficient of 0.97 exceeds the commonly referenced threshold of 0.95. This value (being above the 0.95 threshold) suggests potential item redundancy within the instrument, warranting further refinement in future iterations.

The Cronbach’s alpha for the revised assessment tool was 0.97. While this reflects high internal consistency across domains, this value (being above the 0.95 threshold) suggests potential item redundancy within the instrument, warranting further refinement in future iterations. The final version was implemented in 45 older adults in Nong Phueng Subdistrict, Chiang Mai Province, Thailand.

### 3.4. Pilot Testing

#### 3.4.1. Sociodemographic Characteristics of Participants

The characteristics of the pilot study participants are presented in Table 3. A total of 45 community-dwelling older adults participated in the pilot testing phase, the majority of whom were female (82.22%). Participants were predominantly aged between 60 and 74 years, with the largest proportion in the 70–74-year age group (31.11%). Most participants were married (51.11%) or widowed (35.56%).

More than half of the participants reported no formal education (51.11%), and 60.00% were currently working. Monthly income varied, with the largest proportion reporting an income between 1000 and 3000 Thai baht (35.56%). Most participants lived in single-parent households (71.11%).

Regarding health status, 88.89% of participants reported having at least one chronic health condition. Hypertension (34.19%) and diabetes (27.35%) were the most commonly reported conditions. As participants were allowed to report more than one diagnosis, the total number of reported chronic conditions exceeded the number of participants.

#### 3.4.2. Perceived Age-Friendly Community Characteristics

Among the pilot participants, perceptions of age-friendly characteristics were generally positive across domains and subdomains are summarized in Table 4. These findings reflect only this specific pilot group and should be interpreted as descriptive rather than as a definitive assessment of the community’s overall status. Overall, mean scores across domains fell within the upper range of the response scale, indicating generally high perceived levels of age-friendliness.

Within the Physical and Environmental domain, perceived levels were high for Outdoor Spaces and Buildings (mean = 3.73, SD = 0.91) and Housing (mean = 3.78, SD = 0.95), while Transportation Systems was rated at a moderate level (mean = 3.33, SD = 1.13).

In the Social domain, all subdomains demonstrated high perceived levels, including Social Participation (mean = 3.56, SD = 1.03), Respect and Social Inclusion (mean = 3.93, SD = 0.96), and Civic Participation and Employment (mean = 3.62, SD = 1.13).

Similarly, within the Support and Services domain, participants reported high perceived levels for both Communication and Information (mean = 3.71, SD = 0.82) and Community Support and Health Services (mean = 3.76, SD = 0.91).

In summary, the findings indicate comparatively higher perceived levels in social cohesion and support service domains, whereas relatively lower ratings were observed for transportation-related aspects within this pilot sample. These descriptive results are presented to illustrate variation in perceived age-friendly characteristics captured by the instrument, rather than to provide formal evaluation or benchmarking of community performance.

## 4. Discussion

This study developed and pilot-tested an AFC Assessment Tool designed to capture older adults’ perceptions in a semi-urban Thai community. The adaptation of the response format from a dichotomous checklist to a five-point Likert scale introduced meaningful measurement implications. Likert-type scaling enhances response variability and allows respondents to express gradations in their perceptions, thereby improving discriminatory capacity in community-based assessments [21]. At the same time, polytomous formats may introduce response tendencies—such as central tendency bias or acquiescence—that can affect score distributions independently of the underlying construct [22].

The instrument demonstrated strong content validity and high preliminary internal consistency (α = 0.97), supporting its feasibility for pilot-level use [23,24]. However, several measurement considerations warrant further study. The high reliability coefficient, while positive, may suggest item redundancy or limited heterogeneity. Although this may partly reflect conceptual coherence with the WHO AFC framework, further examination of inter-item correlations and dimensional structure is needed. In addition, clustering of responses at the upper end of the scale suggests a potential ceiling effect, which could limit discriminatory sensitivity across diverse community contexts.

Second, pilot results exhibited clustering of responses toward the upper end of the scale, suggesting a potential ceiling effect. Restricted score variability may limit the instrument’s sensitivity in distinguishing levels of age-friendliness across settings. While these positive perceptions may reflect genuinely favorable conditions in the study community, they may also indicate scale compression or response tendencies that require further investigation.

The three-domain, eight-subdomain structure applied in this study was conceptually derived from the WHO AFC framework [25]. It represents a theoretically based model rather than one empirically confirmed through factor analysis. Due to the modest pilot sample, no factor-analytic procedures were conducted; thus, the proposed structure should be viewed as provisional pending validation in larger samples.

Because this was an exploratory pilot study, construct validity, structural validity, and test–retest reliability were not examined. Future work with larger and more diverse samples is needed to evaluate construct coherence, assess measurement stability, and clarify score distribution patterns, particularly within settings characterized by contextual variability [10].

Pilot implementation in Nong Phueng Subdistrict demonstrated the feasibility of the tool and its ability to identify domain-specific strengths and challenges in a semi-urban community. Interpretation of these findings should consider the demographic characteristics of the pilot sample. The predominance of female participants and lower levels of formal education may have influenced perception-based responses. Prior studies indicate that gender and sociodemographic characteristics influence expectations, social participation, and community engagement through multi-level mechanisms [26]. Thus, the observed perception patterns should be seen as context-specific rather than broadly generalizable.

*Physical and environmental domains.* Higher scores in the housing and outdoor spaces and buildings subdomains suggest favorable perceptions of basic infrastructure and public environments in Nong Phueng Subdistrict. These findings align with the WHO Age-Friendly Cities framework, which emphasizes inclusive design, neighborhood safety, and accessible public spaces as determinants of healthy aging [3,27]. Similar patterns have been reported in other Southeast Asian studies where family-oriented housing and localized infrastructure support aging in place [8,23]. Evidence from Japan further suggests that age-friendly built environments enhance older adults’ well-being, strengthen social connectedness, and support community sustainability by promoting residential continuity [28,29,30,31].

Despite these positive ratings, limitations in physical accessibility—particularly in transportation—may restrict older adults’ social participation. Older adults with lower educational attainment may be especially vulnerable, as limited education is associated with lower health literacy and reduced ability to navigate transportation systems. In Chiang Mai, informal transportation (rod daeng, motorcycle taxis) is widely used but often inconsistent, lacking universal design features, and providing limited coverage in peri-urban areas [32]. These conditions contrast with age-friendly initiatives in high-income countries, where accessible transportation and pedestrian-oriented design are systematically integrated [27,33]. Limited transportation accessibility may reduce opportunities for social, recreational, and civic engagement. A scoping review by Lamanna et al. [34] identified inadequate transportation as a major barrier to social participation among older adults, particularly in rural and resource-limited settings. Consistent with this, transportation accessibility has been positively associated with life satisfaction and social engagement [35], while accessible outdoor environments support social participation and reduce isolation [36].

*Social Domains*. The highest scores were observed in the respect and social inclusion subdomain, indicating a strong sense of social value and inclusion within the community. This finding likely reflects traditional Thai cultural norms that emphasize respect for older persons and intergenerational relationships. Comparable patterns have been reported in other Asian contexts, where cultural values play a central role in shaping social inclusion and social status among older adults [37,38].

Positive ratings in social participation and civic participation and employment further suggest that older adults in this semi-urban community have opportunities to engage in social and civic activities. Prior studies have consistently demonstrated that such opportunities are associated with well-being, quality of life, and healthy aging [30,31]. In the Thai context, social trust, community connectedness, and social inclusion have been identified as important correlates of quality of life among older adults, reinforcing the relevance of social participation as a core component of age-friendly communities [39,40].

*Support and services domains*. High scores in community support and health services and communication and information indicate that local health infrastructure and community-based support systems are accessible and responsive. This finding is consistent with previous studies highlighting the role of primary healthcare networks and community health volunteers in promoting age-friendly environments in Thailand and other Southeast Asian settings [8,9]. Accessible communication and information systems have also been identified as essential components of age-friendly communities, as they enable older adults to navigate services, access support, and maintain independence [3].

From an occupational therapy perspective, the AFC Assessment Tool aligns with the PEOP model, which emphasizes interactions among individuals, environments, and participation in daily life [12]. The tool systematically examines physical, social, and service-related environmental conditions that may facilitate or constrain older adults’ engagement. By centering older adults’ perspectives, the instrument maintains client-centeredness—a core principle of occupational therapy. The identification of strengths (e.g., social inclusion) alongside challenges (e.g., transportation) demonstrates how environmental factors interact with personal and contextual characteristics to influence participation. These insights support community-level environmental modifications, service planning, and intervention development aimed at promoting healthy aging.

Overall, the AFC Assessment Tool provides a context-sensitive means of assessing age-friendliness from older adults’ perspectives and complements administrative or objective assessments. While preliminary psychometric indicators were strong, comprehensive validation is still required. Although the instrument demonstrated strong content validity and high internal consistency in this pilot study, comprehensive psychometric validation has not yet been established. The overall Cronbach’s alpha was high (0.97), which may raise concerns regarding potential item redundancy. While domain-level analyses indicated acceptable internal consistency, the elevated overall alpha may partly reflect conceptual coherence aligned with the WHO age-friendly framework. It is important to note that the three-domain, eight-subdomain structure was theoretically derived rather than empirically established in the present study; therefore, dimensionality, factor loadings, and potential cross-domain item overlap remain unexamined. The preliminary reliability assessment conducted with a small subsample (n = 10) provides only limited insight into internal consistency and should not be interpreted as stable psychometric evidence. Reliability coefficients derived from such a small sample may be sensitive to sampling variability. Nonetheless, structural validity (e.g., exploratory or confirmatory factor analysis), construct validity, and test–retest reliability were not examined due to the limited sample size. Accordingly, future large-scale studies are required to confirm the proposed domain structure through factor-analytic examination, establish construct validity, and strengthen the structural robustness and generalizability of the instrument across diverse settings.

Compared with existing age-friendly assessment tools, the present instrument offers several distinguishing features. Many existing tools rely on administrative indicators and may not capture older adults’ lived experiences. In contrast, this tool is perception-based and tailored to semi-urban settings along the rural–urban continuum. Although clustering of responses at the upper end suggests potential ceiling effects, this may also reflect genuinely favorable perceptions. The clustering of pilot responses at the “High” level suggests a potential ceiling effect, which may limit variability and reduce the instrument’s discriminatory sensitivity. From a measurement perspective, restricted variance can attenuate the ability to detect differences across settings or demographic subgroups. The observed distribution may reflect genuinely favorable environmental perceptions within the study context; however, it may also indicate limited scale differentiation, social desirability tendencies, or homogeneity within the pilot sample.

Furthermore, whereas many age-friendly measurement tools have been validated in high-income urban contexts, this study addresses the relative scarcity of context-sensitive instruments developed within low- and middle-income countries. Semi-urban areas along the rural–urban continuum present unique environmental configurations, including mixed transportation systems, evolving housing patterns, and hybrid social structures. The present tool is specifically adapted to these transitional environments.

Finally, the integration of the PEOP model provides theoretical grounding that extends beyond descriptive environmental assessment. By situating environmental features within a participation-focused occupational framework, the instrument supports interpretation of age-friendliness in terms of functional engagement and daily life performance rather than solely structural adequacy.

Taken together, these elements position the AFC Assessment Tool not as a replacement for existing frameworks, but as a complementary instrument designed to enhance context-sensitive, participation-oriented assessment in semi-urban aging communities.

## 5. Conclusions

This study developed and pilot-validated an AFC Assessment Tool designed to capture older adults’ perspectives within a semi-urban Thai community. The instrument demonstrated strong content validity and high preliminary internal consistency (α = 0.97). While such high reliability may suggest potential item redundancy, the results support the tool’s feasibility for pilot use. The tool is theoretically grounded in the WHO Age-Friendly Cities framework and organized into eight conceptually derived subdomains; however, this structure has not yet been empirically validated through factor analysis and should be considered provisional pending further investigation.

Application of the tool revealed domain-specific strengths, particularly in Housing, Respect and Social Inclusion, and Community Support and Health Services, alongside moderate ratings for Transportation Systems. These patterns underscore the importance of mobility and accessibility in semi-urban settings, where transportation infrastructure may not keep pace with residential and social development.

By incorporating older adults’ perspectives, the AFC Assessment Tool offers a context-sensitive complement to administrative and objective assessments of age-friendly environments. It provides practical utility for community stakeholders and public health professionals seeking to identify priorities and guide age-friendly initiatives in semi-urban communities experiencing demographic aging. Further validation with larger and more diverse samples is needed to confirm the instrument’s dimensional structure and support its broader application across various community settings.

## 6. Implications for Practice and Future Research

The AFC Assessment Tool provides a structured and standardized method for local authorities, urban planners, and healthcare professionals to assess age-friendliness in semi-urban communities from the perspective of older adults. By identifying strengths and barriers across specific domains, the tool can inform evidence-based planning, guide the prioritization of contextually appropriate interventions, and support ongoing monitoring of community readiness for population aging.

Future research should build upon this pilot work through comprehensive psychometric evaluation in larger and more diverse samples. Such efforts are needed to examine test–retest reliability, verify the proposed three-domain structure through CFA, and establish construct validity. Longitudinal studies and cross-community comparisons would further elucidate how age-friendly environments evolve over time and enhance the generalizability of the tool across different settings. Additionally, studies involving more heterogeneous populations are required to assess response distribution patterns and to determine the extent to which potential ceiling effects influence the tool’s discriminatory capacity.

## 7. Limitations

Several limitations should be considered when interpreting these findings. First, the study involved a relatively small sample (n = 45) drawn from a single semi-urban community. Although appropriate for feasibility testing and preliminary psychometric evaluation, this sample size limits generalizability and does not permit advanced analyses such as confirmatory factor analysis.

Second, the demographic composition of the sample—particularly the predominance of female participants and relatively low levels of formal education—may have influenced perception-based responses. Gendered social roles and educational background can shape expectations, access to community resources, and interpretations of environmental support.

Third, because the instrument relies on self-reported perceptions, responses may have been influenced by social desirability or cultural norms that favor positive evaluations of local environments.

Therefore, the findings should be interpreted as exploratory and context-specific, providing an empirical foundation for subsequent validation studies rather than definitive conclusions regarding community age-friendliness.

## Figures and Tables

**Table 1:** Conceptual framework of the AFC Assessment Tool: domains and subdomains derived from the WHO age-friendly cities framework.

Domains	Subdomains	Original Model WHO AFC Framework	Operational Definition & Content (Based on WHO)
Physical & environmental	Outdoor spaces and building	Accessible, safe, and well-maintained parks, public spaces, and buildings.	Older adults’ perceived accessibility, safety, and usability of outdoor spaces and buildings in their community
	Transportation systems	Accessible, affordable, safe, and reliable public transportation for independent mobility	Perceived adequacy and age-friendliness of transportation infrastructure and services
	Housing	Suitable, affordable, and adaptable housing options, supporting ageing in place	Perceived suitability of housing conditions to support safe and independent living
Social	Social participation	Opportunities for social, cultural, and recreational engagement to combat isolation	Older adults’ perceived opportunities for social participation and engagement in community activities
	Respect & social inclusion	Valuing older adults and keeping them integrated into community life	Perceived respect, social inclusion, and age-related attitudes experienced by older adults
	Civic participation & employment	Encouraging continued work, volunteering, and skill-sharing	Perceived opportunities for civic participation, volunteering, and continued contribution to society
Support & services	Communication & information	Easily accessible and understandable information for all.	Perceived accessibility and adequacy of community support services for older adults
	Community support & health services	Access to health, social, and informal care services.	Perceived accessibility and quality of health services relevant to older adults.

**Table 2:** Item distribution and content specification of the AFC Assessment Tool across domains and subdomains.

Domains	Subdomains	Initial Version		Final Version		Rationale for Item Refinement
		Number of Items	IOC Range	Number of Items	IOC Range	
Physical & Environmental	P1: Outdoor spaces and buildings	10	0.4–1.0	9	0.8–1.0	One item was removed due to an IOC value < 0.50. The item was considered ambiguous and conceptually overlapping with other items within the same subdomain.
	P2: Transportation systems	7	0.6–0.8	6	0.8	One item was removed due to an IOC value < 0.50 and redundancy with other items measuring similar constructs.
	P3: Housing	7	0.8–1.0	6	1.0	One item was removed due to an IOC value < 0.50 and conceptual overlap with existing items.
Social	S1: Social participation	6	0.6–1.0	6	0.8–1.0	Items were reworded to improve clarity, readability, and ease of understanding based on expert feedback and cognitive debriefing.
	S2: Respect and social inclusion	8	0.6–0.8	8	0.8–1.0	Items were revised to enhance linguistic clarity and reduce complexity for older adult respondents.
	S3: Civic participation & employment	6	0.4–1.0	5	0.8–1.0	One item was removed due to an IOC value < 0.50 and because it was ambiguous and overlapping with other items in the subdomain.
Support & services	SS1: Communication & information	8	0.6–1.0	8	0.8–1.0	Items were refined to improve clarity and ensure alignment with the operational definition of community support services.
	SS2: Community Support & health Services	4	0.8–1.0	4	1.0	Wording of items was revised to enhance clarity and comprehensibility for the target population.
Total		56	0.4–1.0	52	0.8–1.0	

Note: This table summarizes the item refinement process based on content validity analysis using the IOC. Domains and subdomains were derived from the WHO Age-Friendly Cities (AFC) framework. Operational definitions are presented in Table 1.

**Table 3:** Sociodemographic and health characteristics of pilot study participants (n = 45).

Characteristics		N	Percentage
Gender	Male	8	17.78
	Female	37	82.22
Age (years)	60–64	12	26.67
	65–69	10	22.22
	70–74	14	31.11
	75–79	3	6.67
	Above 80	6	13.33
Marital status	Never married	5	11.11
	Married	23	51.11
	Widowed	16	35.56
	Divorced/Separated	1	2.22
Education	No formal education	23	51.11
	Primary school graduate	9	20.00
	High school graduate	2	4.44
	Some college	8	17.78
	College graduate (bachelor’s degree)	3	6.67
Current working status	Not working	8	40.00
	Working	27	60.00
Income (THB)	No income	2	4.44
	Less than 1000	9	20.00
	1000–3000	16	35.56
	3001–5000	6	13.33
	5001–10,000	2	4.44
	More than 10,000	10	22.23
Family status	Single parent family	32	71.11
	Extended family	13	28.89
Chronic health conditions *	No chronic condition	5	11.11
	Hypertension	20	34.19
	Diabetes	16	27.35
	Dyslipidemia	8	13.67
	Osteoporosis	3	6.67
	Arthritis	3	6.67
	Allergy	3	6.67
	Heart disease	1	1.72

* Participants with chronic health conditions could report more than one diagnosis; therefore, the total number of reported conditions exceeds the number of participants.

**Table 4:** Older adults’ perceptions of age-friendly community characteristics across domains and subdomains.

Domains	Subdomain	Item Code	Mean	SD	Interpretation
Physical & environmental	Outdoor spaces and building	1–9	3.73	0.91	High
	Transportation system	10–15	3.33	1.13	Moderate
	Housing	16–21	3.78	0.95	High
Social	Social participation	22–27	3.56	1.03	High
	Respect & social inclusion	28–35	3.93	0.96	High
	Civic participation & employment	36–40	3.62	1.13	High
Support & services	Communication & information	41–48	3.71	0.82	High
	Community support & health service	49–52	3.76	0.91	High

Note: Results reflect perceived age-friendliness and are not intended for benchmarking or population-level comparison.

## Data Availability

The minimal dataset underlying the results is available in the Figshare repository at https://doi.org/10.6084/m9.figshare.31149106.
